# Development of an exercise programme for balance abilities in people with multiple sclerosis: a development of concept study using Rasch analysis

**DOI:** 10.1186/s40945-021-00120-3

**Published:** 2021-12-15

**Authors:** Karl Martin Sattelmayer, Odile Chevalley, Jan Kool, Evelyne Wiskerke, Lina Nilsson Denkinger, Katia Giacomino, Emmanuelle Opsommer, Roger Hilfiker

**Affiliations:** 1 School of Health Sciences, HES-SO Valais-Wallis, Leukerbad, Switzerland; 2 School of Health Sciences (HESAV), University of Applied Sciences and Arts Western Switzerland (HES-SO), Lausanne, Switzerland; 3 Rehazentrum Valens - Kliniken Valens, Taminaplatz 1, 7317 Valens, Switzerland; 4 Department of Rehabilitation Sciences, KU Leuven - University of Leuven, Tervuursevest 101, 3001 Leuven, Heverlee Belgium

**Keywords:** Postural balance, Multiple sclerosis, Exercise, Exercise therapy, Walking, Exercise difficulty

## Abstract

**Background:**

People with multiple sclerosis (PwMS) frequently have impaired balance from an early stage of the disease. Balance difficulties can be divided into categories; although, to date, these lack scientific foundation. Impaired balance in PwMS can be addressed using specific and challenging exercises. Such exercises should provide an optimal challenge point; however, the difficulty of balance exercises is often unknown, making it difficult to target the exercises to an individual’s abilities.

The aims of this study were: to develop an exercise programme for PwMS relating the exercises to the balance problem categories; to establish the order of difficulty of exercises in each category and; to evaluate the content and structural validity of the exercise programme.

**Methods:**

A “construct map” approach was used to design and develop an exercise programme for PwMS. Potentially relevant balance exercises were identified, then a framework was set up, comprising four dimensions (subsequently reduced to three dimensions) of balance exercises. The relevance, comprehensibility, and comprehensiveness of the exercise programme were rated by 13 physiotherapists, who also linked 19 key exercises to balance categories. A total of 65 PwMS performed the 19 balance exercises, rated their difficulty and commented on the relevance and comprehensibility of each exercise. A Rasch model was used to evaluate the relative difficulty of the exercises. To assess fit of the data to the Rasch model a rating scale model was used, which is a unidimensional latent trait model for polytomous item responses.

**Results:**

Evaluation by the physiotherapists and PwMS indicated that the content validity of the exercise programme was adequate. Rasch analysis showed that the latent trait “balance exercises in PwMS” comprised three subdimensions (“stable BOS”, “sway” and “step and walk”). The 19 balance exercises showed adequate fit to the respective dimensions. The difficulties of the balance exercises were adequate to cover the ability spectrum of the PwMS.

**Conclusion:**

A balance exercise programme for PwMS comprising three dimensions of balance exercises was developed. Difficulty estimates have been established for each of the exercises, which can be used for targeted balance training. Content and structural validity of the programme was adequate.

**Supplementary Information:**

The online version contains supplementary material available at 10.1186/s40945-021-00120-3.

## Introduction

People with multiple sclerosis (PwMS) frequently have balance problems and an increased risk of falling [1]. A meta-analysis of individual patient data by Nilsagård et al. [2] found that 56% of PwMS had one fall within 3 months and 37% had at least two falls. Falls are associated with fractures [3], fear of falling [4] and reduced quality of life [5].

Risk factors for falling are primary progressive multiple sclerosis (MS) and higher disease severity, evidenced by higher scores on the Expanded Disability Status Scale (EDSS) [2]. Other authors [6] have identified continence issues, previous falls history or prescribed medications (e.g. muscle relaxants) [7] as risk factors. In addition, Gunn and colleagues [5] found that cognitive impairments, use of mobility aids and altered balance are risk factors for falling.

Impaired balance abilities in PwMS contribute to an increased fall risk [8]. For example, a study by Mazumder et al., [9] found that 43% of falls were caused by loss of balance. Balance problems [10] are more severe in primary progressive MS and secondary progressive MS than in relapsing-remitting MS [11]. Postural responses to balance disturbances are very slow compared with other neurological conditions [12]. Balance disabilities increase during the course of MS, but can be measured even in individuals with mild levels of disability [12] or recent disease onset [13].

Some authors state that problems with balance control in PwMS fall into three interrelated categories [8, 10]. The first category “decreased ability to maintain position” relates to the finding that PwMS show increased body sway and sway velocity in quiet stance with open eyes compared with healthy people. Body sway increases when participants close their eyes [14]. Secondly, PwMS have “limited and slowed movements towards limits of stability”. This is observed in clinical tests, such as the functional reach test [13] or the step test [11]. The third category “delayed responses to postural displacements and perturbations” impairs the individual’s ability to react to unpredictable circumstances during activities of daily living (ADL), such as walking on unstable surfaces or walking in crowded places. However, since balance responses to perturbation might differ in ambulation compared with non-ambulation, this last category may consist of two different categories. It is therefore not certain whether a balance training programme for PwMS should comprise three or four different balance categories.

If balance skills are not a unidimensional construct, a specific treatment programme for balance disorders must include balance exercises in order of increasing difficulty within each category. Health professionals can only design exercises that follow a consistent progression when it is known which exercise belongs to which balance category. For example, if an individual with MS has problems in the balance category “decreased ability to maintain position”, exercises from this category should be selected and exercises from other categories are unlikely to improve these specific balance problems as effectively.

Interventions that aim to decrease balance impairments are recommended for fall prevention in PwMS [8]. Several balance exercise programmes for PwMS have been proposed. A recent systematic review [15] identified five randomized controlled trials (RCTs) investigating gait, balance and functional training in PwMS. Three of the studies prescribed group exercise programmes [16–18], one study reported a home-based balance training [19], and one study used an individual balance training distinguishing between motor and sensory strategy training [20].

In addition, evidence suggests that physiotherapy interventions may enhance balance in this patient population [21, 22].

When balance exercises are prescribed it is difficult to target the exercise difficulty to the abilities of the PwMS. The key to effective motor learning is to identify the optimal challenge point for a learner [23]. Guadagnoli et al. used the following definition of the optimal challenge point: “the optimal challenge point represents the degree of functional task difficulty an individual of a specific skill level would need in order to optimize learning.” ( [23] p216) Thus, if balance exercises are not challenging enough or too challenging, the balance abilities of PwMS will not improve sufficiently. One important element (among others such as motivation or the focus of attention [24]) of the prescription process is that health professionals adjust the difficulty of the exercise to the abilities of the individual with MS. However, the difficulty of balance exercises has not yet been systematically established, in contrast to the difficulty of balance tests (e.g. [25]). Targeting of exercise difficulty to participant ability is therefore frequently based on assumptions and pragmatic reasoning. A systematic classification of the difficulty of balance exercises would support health professionals in their decision-making process, enabling them to find the optimal challenge point for individual patients.

The aims of this study were therefore: to develop an exercise programme for PwMS, relating exercises to balance problem categories; to establish the order of difficulty among exercises in each category and; to evaluate the content and structural validity of the programme.

## Methods

The study involved the following stages: a large pool of balance exercises was established, from which to select key exercises that could be performed within a one-hour session. The content validity of the key exercises was examined in an expert round comprising experienced physiotherapists. The structural validity of the balance exercise programme was analysed in a cross-sectional study, which included analysis of unidimensionality, fit of the balance exercises to the Rasch model, balance exercise difficulty, and assessment of whether the programme covered the balance ability spectrum of PwMS.

A “construct map” approach [26] was used to design and develop the exercise programme for PwMS. This approach comprises four steps: “construct map”, in which the construct to be measured is defined; “item design” balance tasks that range from easy to difficult are designed, with the aim of covering the whole spectrum of PwMS balance abilities; “outcome space”, in which a scoring system for self-reported balance task difficulty is developed and; “measurement model”, in which empirical self-reported difficulties are mapped to the theoretical model of the construct under investigation (i.e. balance abilities in PwMS). In other words, the final step evaluates how well the observed data fit to the postulated measurement model.

### Step 1: construct map

A literature search was carried out for balance categories and balance dimensions. A model proposed by Pai et al. [27] was used as a guide for the design of the balance exercise dimensions.

### Step 2: item design

The literature (books, websites, scientific literature) was reviewed for potentially relevant balance exercises and a pool of 98 balance exercises was set up. Three reviewers (LNB, RH, KMS) with expertise in exercise in PwMS searched online bookstores for books on exercises and MS. We searched PubMed and Scholar Google for publications reporting on exercise programmes in PwMS. In addition, we searched books and scientific articles on falls prevention, and this search was not limited to PwMS in order to increase the number of potential eligible exercises. We did not perform a systematic search such as recommended for systematic reviews. Exercises were selected based on i) feasibility aspects (i.e. devices are not needed to perform the exercise at home), ii) safety aspects (i.e. the exercises can be performed without supervision) and iii) environmental aspects (i.e. the exercises can be performed at home).

Six physiotherapists, who had several years of experience treating people with neurological diseases, were asked to estimate how many exercises could be evaluated in PwMS within a one-hour session. This time limit was chosen for pragmatic reasons (assuming one session of physiotherapy and a potentially limited period of concentration for some PwMS). The physiotherapists considered that fewer than 20 exercises was a feasible number. Therefore, in collaboration with the six physiotherapists, 19 balance exercises were selected, and classified as “key” exercises, which could subsequently be modified to make them easier or more difficult.

The 19 key exercises were submitted to a larger group of 13 physiotherapists. The criterion for selection of the physiotherapists was experience in working with PwMS. The physiotherapists allocated the 19 exercises to the four balance dimensions (i.e.” stable base of support (BOS)”, “sway”, “step” and “walk”). They could also add a new category (i.e. balance dimension) or modify the existing categories. In addition, the physiotherapists ordered the balance exercises from easy to difficult and commented on the relevance of the exercises, their comprehensiveness (i.e. were key exercises missing within the balance dimensions), and the comprehensibility of the instructions and scoring options. The online platform OptimalSort [28] was used for data collection. More detailed information regarding the physiotherapists’ instructions are available as a video here [29].

Agreement regarding the allocations and rankings of the exercises were analysed using Cohen’s kappa and the similarity matrix of the OptimalSort programme, in which the number of times a pair of exercises were grouped together and the percentage of participants agreeing with an exercise pairing are shown. This matrix shows the exercises where grouping in the same dimension was less clear. In addition, the physiotherapists were asked whether the exercise instructions were adequate.

### Step 3: outcome space

A self-reported six-point rating scale (i.e. rated by the participant) was used to measure the difficulty of the balance exercises (where 5 = “very easy”; 4 = “easy”; 3 = “challenging”; 2 = “very challenging”; 1 = “too challenging, it is almost dangerous” and; 0 = “dangerous, I would or could fall”, or the participant did not perform the exercise. This scale was based on a rating scale used in previous research [30] and adapted based on Kent [31] to reflect abilities rather than deficits.

### Step 4: measurement model – evaluation of the structural validity of the balance exercise programme

#### Participants

PwMS were recruited from different sites and settings in Switzerland; from private practices in Valais and Vaud, and from the rehabilitation clinic Valens. Data were collected between October 2018 and April 2020.

Inclusion criteria were: able to walk a minimum of 20 m independently, with or without the use of walking aids; able to stand for more than 3 s without help or aids; diagnosed with MS by a medical doctor; able to understand and execute project-related instructions (project, exercise, etc.); allowed to perform exercises (i.e. a prescription for active physiotherapy). Potential participants were assessed by project collaborators. After providing informed consent to participate in the study, all subjects were asked to perform a set of 19 key balance exercises under the supervision of a trained physiotherapist. The physiotherapist showed each participant written instructions and a photograph of the exercise, in the same way as would be provided for a home-based exercise programme (Additional file 1). If the instructions were not understood, the physiotherapist demonstrated and explained the exercise. Before performing the exercise participants were asked to rate the perceived risk of falling. They could decline to perform the exercise because of safety reasons. Each exercise was performed for 15 s. The physiotherapist remained close to the participant to ensure safety. Immediately after performance of each balance exercise, participants were asked to rate the difficulty of the exercise, and to comment on the relevance and comprehensibility of the exercise, exercise instructions and response options. Study data were collected and managed using REDCap, an electronic data capture tool hosted at HES-SO [32, 33]. The study was approved by the responsible ethics committees, in Vaud and St. Gallen, Switzerland (ID 2018–00824).

##### Sample size

In order to achieve stable item calibrations within ½ logits the minimal sample size for the study was set at 64 participants [34].

#### Data analyses

Data were analysed using the statistical programme Winsteps (4.5.5) [35]. A rating scale model [36] was used, which is a unidimensional latent trait model for polytomous item responses. The difficulty of each exercise and the ability of each participant are reported in logits (i.e. log of the odds). Higher logits indicate higher exercise difficulty or higher participant ability. A Rasch model including all exercises in one overarching dimension was compared with a model comprising three dimensions (“stable base of support”, “sway” and “step and walk”), and with a model comprising four dimensions (“stable base of support”, “sway” and “step” and “walk”).

##### Unidimensionality

Unidimensionality of the data was explored using principal component analysis (PCA) of the standardized residuals from the Rasch analysis [37]. A threshold of 2 eigenvalues was used to indicate the presence of a potential secondary latent trait within the analysis [38]. In addition, contrast plots were searched for clusters of exercises that could represent a separate latent trait. Disattenuated correlations between clusters of exercises were calculated and interpreted as follows: correlations > 0.7 indicated that clusters measured the same latent trait. Correlations < 0.3 indicated that they measured different latent traits.

##### Local dependency

Local dependency of items was analysed using Yen’s Q3 statistic (correlation of the raw residuals) with a critical value of 0.3 [39]. To be able to meaningfully analyse local dependency it has been suggested that at least 20 items should be available per analysis [40] and a variety of critical values have been reported ranging from 0.1 to 0.7. There is limited evidence for the validity of the critical values and they are not sensitive to the specific characteristics of the data [39].

##### Item fit

Item fit of the exercises to the rating scale model was assessed using a guideline reported by Linacre [41]. First, the data were checked for negative point biserial correlations, which may indicate problems with response level scoring. Secondly, fit of the items was assessed using outfit mean-square statistics, which have a chi-square distribution with an expected value of 1 [42]. A range between 0.5 and 1.7 is considered as sufficiently valid for clinical observations in the Rasch literature [43]. Mean-square statistics are relatively independent of sample size for polytomous data compared to t-statistics [44]. In addition, outfit mean-square values produce less Type 1 errors when a rating scale model is used compared to infit-mean square values [44].

##### Person and item separation reliability

The person and item separation reliability were analysed using the separation indices and the Rasch reliability coefficients. For person separation reliability, values > 2 are considered good for the separation index and > 0.8 for the reliability coefficient [45]. For item separation reliability, values > 3 are considered good for the separation index and > 0.9 for the reliability coefficient [45].

##### Item thresholds and targeting

The difficulty of each response option for the balance exercises (i.e. scores 0, 1, 2, 3, 4 or 5) was explored using Rasch-Thurstone thresholds, which indicate the ability rating of a participant who has a 50% chance of scoring in the response option above or below the threshold [46]. A second step analysed whether the targeting of the balance exercises was adequate to measure the latent trait (i.e. balance ability in PwMS). The range of item difficulties should cover the whole spectrum of participants’ abilities. A Wright map was used to visualize the targeting of the exercises [47]. To analyse category disordering, the average difficulty measure for each category was analysed as recommended by Linacre [48] (i.e. it was inspected if they increased monotonically across categories).

##### Correlation between expert round difficulty ranking and Rasch measure

To further triangulate our results, we calculated the correlation of the mean rank position of the exercises (from the expert rounds) within each dimension with the Rasch estimate for each exercise.

## Results

### Step 1: construct map

A framework consisting of four dimensions of balance exercises was set up, as follows:

1. Stable base of support (BOS) and ability to centralize the centre of mass (CoM): termed “*stable BOS*”.
2. Voluntary movement or shift of the CoM towards the limits of stability: termed “*sway*”.
3. Voluntary movement of the CoM over the limits of stability and creation of a new BOS: termed “*stepping*”.
4. Controlling the CoM during a steady state movement: termed “*walking*”.

**Fig. 1:**
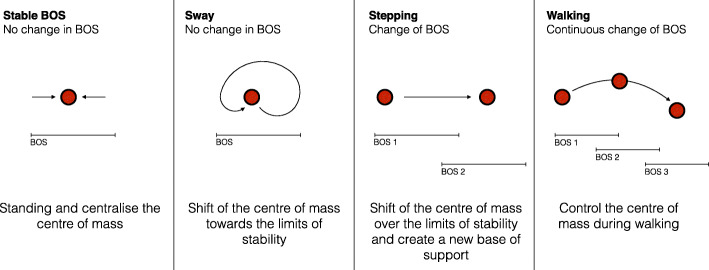
Four dimensions of balance exercises in people with multiple sclerosis (PwMS). BOS: base of support

The initial construct map is shown in Fig. 1.

### Steps 2 and 3: item design and outcome space

The initial pool of 98 exercises is shown in Additional file 2, and the 19 key exercises are shown in Additional file 3.

The expert round comprised 13 physiotherapists; 11 from Switzerland, one from Germany and one from Austria. All participants had considerable work experience as physiotherapists (median 12 years; interquartile range (IQR) 5–18). The median number of PwMS treated per year by each physiotherapist was 5 (IQR 3.5–120). The characteristics of the physiotherapists in the expert round are shown in Table 1.

**Table 1:** Characteristics of the 13 physiotherapists in the expert round

Characteristics	
Place of work, *n* (%)	
Austria	1 (8%)
Germany	1 (8%)
Switzerland	11 (84%)
Age, years, median (IQR)	33 (31–40)
Experience as physiotherapist, years, median (IQR)	12 (5–18)
Sex, *n* (%)	
Women	9 (69%)
Men	4 (31%)
People with MS per year, *n*, median (IQR), range	5 (3.5–35), 2-120

*MS* multiple sclerosis, *IQR* interquartile range

**Fig. 2:**
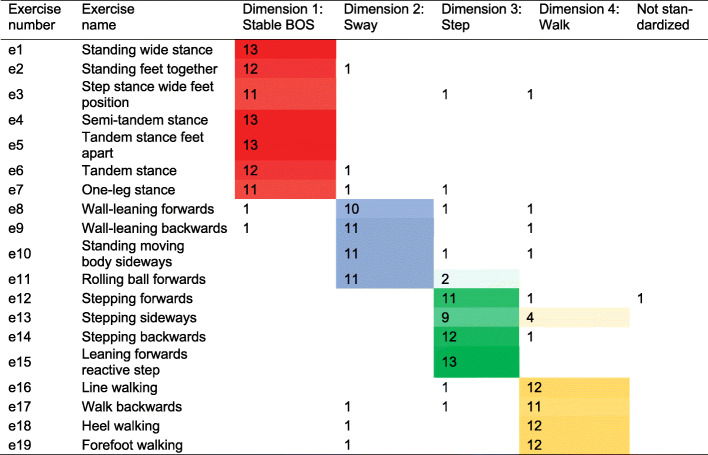
Standardization grid showing the distribution of physiotherapists’ classifications across the balance dimensions

Chance-corrected agreement between physiotherapists’ classification of balance dimensions (kappa) was 0.73, with a 95% confidence interval (95% CI) of 0.65–0.81. The majority of physiotherapists classified seven exercises into dimension 1 and four exercises into each of the remaining dimensions. All ratings are shown in a standardization grid in Fig. 2. For most exercises, the percentage of participants who agreed with each card pairing within each dimension was high. However, three exercises had a lower agreement with other exercises within their classified dimension. These were “stepping sideways”, “rolling ball forwards” and “heel walking”. The percentage of participants who agreed with each card pairing is shown in Additional file 4. The most difficult exercises within each balance dimension were: “one-leg stance”, with a mean rank position of 6.9 within dimension 1; “wall-leaning backwards”, with a mean rank position of 3.5 within dimension 2; “leaning forwards reactive step” with a mean rank position of 3.3 within dimension 3; and “walk backwards” with a mean rank position of 3.2 within dimension 4. All balance exercises and the rating of their balance dimension and corresponding difficulty position are shown in Additional file 3.

Colours were used as follows: red for dimension 1, blue for dimension 2 and green for dimension 3. Colour shading was used to illustrate the frequency of classifications (light: not frequent; dark: frequent). All exercise descriptions are presented in Additional file 3.

#### Expert round regarding content and face validity

The physiotherapists’ expert assessment of the comprehensiveness of the proposed balance exercises did not mention any important missing exercises in each dimension. Regarding face validity, it was discussed that the exercises in the “stable BOS” dimension could already involve shifting the centre of the mass towards the limits of stability; for example, when a person with low balance ability stands still without support, they may already have explored their limits of stability, even though this is not included in the exercise instruction. Furthermore, the physiotherapists considered that some exercises might train different constructs, depending on the impairments of PwMS. Making rankings of difficulty was perceived as difficult by the physiotherapists. Several instructions were amended after the expert round, in order to improve their comprehensibility.

#### PwMS perspective on the exercises

Regarding the relevance and comprehensibility of the exercises, exercise instructions and response options, a number of following points were mentioned by the PwMS, as follows.

##### Relevance

Participants reported that all exercises, with the exception of two (stepping sideways [exercise e13], wall-leaning backwards [exercise e9]) contained elements that they used in ADL.

##### Comprehensibility

There were several exercises in which the photographs and instructions were not clear, especially exercises e12 to e15 (a step was required). A general remark was that for dynamic exercises one picture was insufficient to show the whole movement sequence. This applied mainly to the instructions and depiction of how to perform the exercise safely (i.e. where to perform the exercises or what objects, such as tables, chairs, sofas, should be used to increase safety). Suggestions to improve this were to perform exercises in a corner or next to stable objects.

Furthermore, some PwMS found it difficult to differentiate between the response options “very easy” and “easy”, as well as between “too challenging, it is almost dangerous” and “dangerous, I would or could fall”.

### Step 4: measurement model – evaluation of the structural validity of the balance exercise programme

#### Overview of the sample

A total of 66 PwMS were considered potentially eligible for this study. Data were lost for one PwMS due to technical problems. Therefore, data for 65 participants were included into the analysis. The study sample consisted of considerably more women (*n* = 48; 74%) than men. The median age of participants was 53 years (IQR 47–58). Relapsing-remitting MS was the most common type in the study sample (*n* = 30). The median EDSS score was 5 (IQR 3.5–6). The characteristics of the study participants are shown in Table 2.

**Table 2:** Participants’ characteristics

Characteristics	
Sex, *n* (%)	
Women	48 (74%)
Men	17 (26%)
Total	65
Age, years, median (IQR), range	53 (47–58), 24–78
German speaking, *n* (%)	35 (54%)
French speaking, *n* (%)	30 (46%)
Relapsing-remitting MS, *n* (%)	30 (46%)
Primary progressive MS, *n* (%)	12 (18%)
Secondary progressive MS, *n* (%)	18 (28%)
Type not known, *n* (%)	5 (8%)
EDSS, median (IQR)	5 (3.5–6)

*MS* multiple sclerosis, *EDSS* Expanded Disability Status Scale, *IQR* interquartile range

#### Data analyses

Initial analysis was performed using all exercises within one Rasch model. The analysis showed that unidimensionality could not be confirmed with PCA of residuals. An eigenvalue of 3.1 was above the predefined threshold of 2 for unidimensionality. In addition, item fit measured with mean-square statistics found that four exercises showed misfit to the rating scale model (e3, e9, e18 and e17). These findings suggest that the latent dimension (i.e. balance abilities in PwMS) consisted of several relevant subdimensions. In a second step a Rasch model consisting of three dimensions (i.e. “stable base of support”, “sway” and “step and walk”) was compared with a model consisting of four dimensions (i.e. “stable base of support”, “sway”, “step” and “walk”). The fit measures indicated that the three-dimension solution was better.

The final model for the Rasch analysis therefore comprised the following three dimensions:

- Dimension 1: exercises in which participants had a stable base of support and were required to centralize their centre of mass, e.g. during standing (termed “stable BOS”). Exercises e1–7 were classified into dimension 1.
- Dimension 2: exercises in which participants had to voluntarily move their centre of mass towards their limits of stability, e.g. during swaying (termed “sway”). Exercises e8–11 were classified into dimension 2.
- Dimension 3: exercises in which participants had to voluntarily move their centre of mass over their limits of stability and create a new base of support, e.g. during stepping or walking (termed “step and walk”). Exercises e12–19 were classified into dimension 3.

##### Unidimensionality

PCA of residuals confirmed unidimensionality of all three dimensions; i.e. eigenvalues were below the threshold of 2 (i.e. dimension 1: 1.9; dimension 2: 1.9; dimension 3: 1.9). For all dimensions, the observed variance approximated the expected variance of the PCA. Disattenuated correlations for all three dimensions were > 0.7. The smallest disattenuated correlation for each dimension was: 0.83 (dimension 1), 1 (dimension 2) and 0.95 (dimension 3) (Table 3).

**Table 3:** Analysis of dimensionality

Dimension	Eigenvalues in first contrast	Disattenuated correlation
Dimension 1	1.9	Item cluster (1–3): 0.83 Item cluster (1- 2): 1.0 Item cluster (2- 3): 1.0
Dimension 2	1.9	Item cluster (1–3): 1.0
Dimension 3	1.9	Item cluster (1–3): 0.95

##### Local dependency

In dimension 1, four item pairs had correlations above 0.3. (e6-e7: − 0.48; e5-e7: − 0.4; e2-e5: − 0.35; e4-e7: − 0.31). Within dimension 2, four pairs of items had correlations above 0.3 (e8-e10: − 0.72; e9-e11: − 0.64; e9-e10: − 0.46; e8-e11: − 0.45). In dimension 3, five item pairs had correlations above 0.3 (e16-e17: 0.54; e15-e18: − 0.43; e13–19: − 0.38; e16–19: − 0.37; e12–18: − 0.32).

##### Item fit

**Fig. 3:**
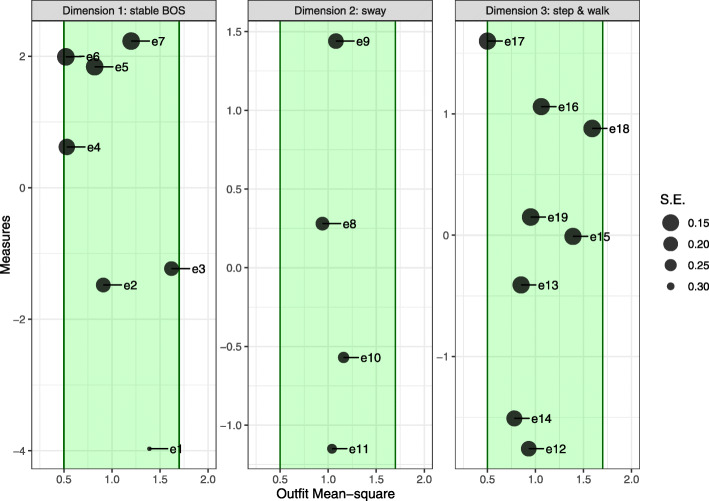
Bond and Fox pathway map for three dimensions of balance in PwMS. The measure (difficulty estimate) of each balance exercise is plotted against its outfit mean-square statistic. The size of points represents the inverse of the standard error (S.E.) of the difficulty estimate. Green areas indicate adequate fit to the Rasch model

All balance exercises in dimension 1 (“stable BOS”) showed adequate mean-square statistics. Fit values ranged between 0.52 (e6: tandem stance) and 1.63 (e3: step stance feet wide apart). Exercises in dimension 2 (“sway”) showed nearly perfect fit with values close to 1. Similar findings were observed for dimension 3 (“step and walk”). Fit statistics ranged between 0.5 (e17: walk backwards) and 1.59 (e18: heel walking). Fit statistics are shown in Fig. 3 and Table 4.

**Table 4:** Overview of the difficulty and fits statistics of the balance exercise in people with multiple sclerosis (PwMS)

Exercise Id	Exercise name	Dimension	Exercise difficulty in logits	S.E.	Outfit Mean-square	Point biserial correlation
e1	Standing wide stance	Stable BOS	−3.97	0.31	1.39	0.52
e2	Standing feet together	Stable BOS	−1.48	0.2	0.91	0.76
e3	Step stance wide feet position	Stable BOS	−1.23	0.2	1.62	0.67
e4	Semi-tandem stance	Stable BOS	0.62	0.16	0.53	0.87
e5	Tandem stance feet apart	Stable BOS	1.84	0.13	0.82	0.78
e6	Tandem stance	Stable BOS	1.99	0.13	0.52	0.8
e7	One leg stance	Stable BOS	2.23	0.13	1.2	0.72
e8	Wall leaning forwards	Sway	0.28	0.22	0.94	0.77
e9	Wall leaning backwards	Sway	1.44	0.18	1.08	0.75
e10	Standing moving body sidewards	Sway	−0.57	0.26	1.16	0.67
e11	Rolling ball forwards	Sway	−1.15	0.28	1.04	0.67
e12	Stepping forwards	Step and walk	−1.76	0.18	0.93	0.71
e13	Stepping sidewards	Step and walk	−0.41	0.14	0.85	0.83
e14	Stepping backwards	Step and walk	−1.51	0.17	0.78	0.78
e15	Leaning forwards reactive step	Step and walk	−0.01	0.14	1.39	0.76
e16	Line walking	Step and walk	1.06	0.14	1.06	0.82
e17	Walk backwards	Step and walk	1.6	0.14	0.5	0.81
e18	Heel walking	Step and walk	0.88	0.13	1.59	0.74
e19	Forefoot walking	Step and walk	0.15	0.13	0.95	0.83

*S.E.* standard error, *BOS* base of support

##### Person and item separation reliability

Person reliability: Dimension 1 had a person separation index of 2.75 and a reliability of 0.88. For dimension 2, a person separation index of 1.75 and a reliability of 0.75 were found. Dimension 3 had a person separation index of 2.78 and a reliability of 0.89.

Item reliability: Dimension 1 had an item separation index of 10.17 and a reliability of 0.99. For dimension 2, an item separation index of 3.18 and a reliability of 0.91 were found. Dimension 3 had an item separation index of 7.26 and a reliability of 0.98.

##### Item thresholds and targeting

Within dimension 1, the difficulty estimates ranged between − 3.97 (e1: standing wide stance) and 2.33 (e7: one-leg stance) logits. The difficulty in dimension 2 ranged between − 1.15 (e11: rolling ball forward) and 1.44 (e9: wall-leaning backwards) logits. Within dimension 3, the difficulty ranged between − 1.76 (e12: stepping forwards) and 1.6 (e17: walk backwards) (Table 4).

**Fig. 4:**
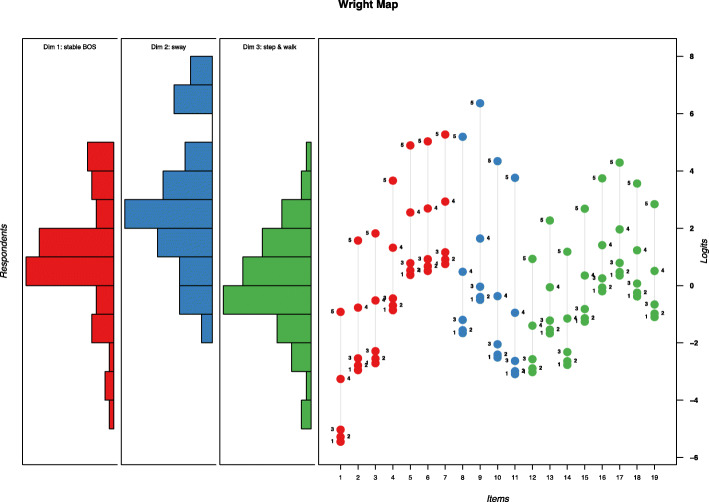
Wright Map. On the left-hand side the abilities of the participants are plotted as histograms. On the right-hand side the Rasch-Thurstone thresholds for the 19 key exercises (Items) are visualized. Note: The three dimensions (Dim.) were analysed separately. Therefore, it is not possible to compare the difficulties of exercises across dimensions. Colours were used as follows: red for dimension 1, blue for dimension 2 and green for dimension 3

Targeting of exercises in dimension 1 was adequate. Ability estimates for participants in dimension 1 ranged between −4.41 and 4.91 logits. Exercise e1 (standing wide stance) was analysed as having the lowest Rasch-Thurstone threshold with −5.45 logits (threshold 1, which is the threshold between a score of 0 and 1) (Fig. 4). The highest Rasch Thurstone threshold was analysed for exercise e7 (one-leg stance) with a logit estimate of 5.27 (threshold 5, which is the threshold between a score of 4 and 5). The abilities of participants ranged between −1.69 and 7.82 logits in dimension 2. The lower end of the ability spectrum was sufficiently covered, with the lowest threshold being −3.09 logits (exercise e11; threshold 1). However, the upper end of the ability spectrum was not fully covered, with the highest threshold being 6.36 (exercise e9; threshold 5). Within dimension 3, the ability estimates of participants ranged between − 4.14 and 4.12 logits. The smallest Rasch-Thurstone threshold was analysed for exercise e12 (− 3.02 logits; threshold 1) and the highest for exercise e17 (4.29 logits; threshold 5). Therefore, targeting to the lower spectrum of abilities was not optimal in this dimension. Within dimension 1, we observed 1 category disordering (i.e. item e5 category 2 and 3 were slightly disordered). No category disordering occurred in dimension 2. In dimension 3, a category disorder was found in items e16 (categories 0 and 1) and e18 (categories 3 and 2). The results for all categories are presented in Additional file 5.

##### Correlation between expert round difficulty ranking and Rasch measure

The correlation between the mean rank position (i.e. expert round ranking) and Rasch measure was high for dimension 1 (r: 0.92; p:0.003), dimension 3 (r: 0.8; p: 0.2) and dimension 4 (r: 0.92; p: 0.08). However, the correlation was only moderate in dimension 2 (r: 0.49; p: 0.51). All correlations are presented in the Additional file 6.

##### Adverse events

No adverse events were reported during this study.

##### Sensitivity analysis

To test whether the fit to Rasch model increased when disordered categories were collapsed a sensitivity analysis was performed. In dimension 1 “Stable BOS” item e5 showed a disordering in categories 1 and 2. In dimension 3 “Step and walk” disordered categories were observed for items e16 (categories 0 and 1) and e18 (categories 2 and 3). Categories were collapsed in these items and the analysis was repeated. To determine whether the fit to the Rasch model increased we compared the AIC (Akaike Information Criterion) and BIC (Bayesian Information Criterion) of both models. For dimension 1, both global fit indices showed that the fit to the Rasch model did not improve when the categories were collapsed. For dimension 3, the results were inconclusive. The AIC showed a better fit when categories were collapsed, and the BIC showed a better fit for the model with non-collapsed categories (Table 5).

**Table 5:** Sensitivity analysis

**Dimension 1**	**AIC**	**BIC**
Model with non-collapsed categories	914.51	1223.37
Model with collapsed categories	914.55	1300.19
**Dimension 3**		
Model with non-collapsed categories	1126.18	1438.51
Model with collapsed categories	1097.03	1519.09

*AIC* Akaike Information Criterion, *BIC* Bayesian Information Criterion

Because 3 of the 4 fit analyses indicated a better fit to the Rasch model when categories were not collapsed, we kept the analysis with non-collapsed categories as primary analysis.

## Discussion

This study developed a balance exercise programme for PwMS, based on an expert round of 13 experienced physiotherapists and a study group of 66 PwMS. The three main findings of the study are as follows: first, an expert round successfully established the content validity of the proposed balance dimensions and the allocation of 19 key balance exercises to these dimensions. Secondly, fit to the Rasch model was adequate if dimension 3 “stepping” and dimension 4 “walking” were combined, since, together, these two dimensions formed an overarching unidimensional construct. Thirdly, the difficulties of the balance exercises were adequate to cover the ability spectrum of the PwMS (i.e. adequate targeting). In summary, it was possible to create a balance programme for the proposed balance dimensions “stable BOS”, “sway” and “step and walk”.

Regarding the content validity of the proposed set of balance exercises, the exercises were explored from the perspective of health professionals and of PwMS, as proposed by the Cosmin group [49, 50]. The study included professionals with considerable experience in prescribing exercises for PwMS. Only physiotherapists were included in the expert round, as this group was considered to be particularly relevant for prescribing balance exercises for PwMS. Evidence for adequate content validity was analysed regarding relevance (i.e. the included exercises were relevant for the respective balance dimensions and the population of interest), comprehensiveness (i.e. key exercises for each balance dimension were integrated), and comprehensibility (i.e. PwMS and health professionals rated instructions and response options as understandable). The perspective of PwMS regarding the exercises was evaluated after each exercise performance, and participants provided feedback on the clarity of the exercise description and instructions, safety aspects, balance exercise, image quality and clarity, and the possibility of performing the exercise at home and adapting the exercise to their home surroundings. The perspective of PwMS will be used to adapt the balance programme within the categories reported above.

Rasch analysis found that the item fit of all 19 balance exercises to the rating scale model was adequate; i.e. no balance exercise was excluded based on the item fit statistics. However, this was only seen when the balance dimensions were analysed separately. Therefore, every balance exercise provides information about the respective latent dimension. Similarly, the assumption of unidimensionality was fulfilled only when the balance exercises were analysed in three separate latent dimensions. When all exercises were analysed together the PCA of residuals exceeded the threshold of 2 eigenvalues, which was used as an indicator for unidimensionality. From a clinical point of view, the separation of balance exercises into distinct dimensions is supported by evidence; e.g. Shumway-Cook and Woollacott [51] proposed a systems model of postural control, which emphasizes that postural control for stability and orientation requires a multitude of neural and musculoskeletal systems. Therefore, the dimensions of balance exercises in the current study require a similar interaction of postural control systems within each of the balance dimensions. Furthermore, PwMS with balance problems can increase their balance abilities within each dimension separately, making targeted training possible.

In addition, the proposed latent traits of the balance exercises in the current study are very similar to the three categories of balance problems proposed by Cameron and Nilsagard [10]. The first two traits are almost identical. Only the category “delayed responses to postural displacements and perturbations” differs slightly from the current proposed latent trait “step and walk”, because, in order to design a programme that could be carried out at home or with minimal external help, external perturbations were not included in the balance programme in the current study. However, all the exercises in the third category require participants to control their balance during postural displacements.

Other authors have analysed unidimensionality of balance tests and have confirmed unidimensionality over a wide range of balance tests, which are similar to the balance exercises used in the current study. For example, La Porta et al. [25] reported that 12 of the Berg Balance Scale items showed unidimensionality when evaluated in samples of different aetiologies in neurological rehabilitation. A further example is a study by Franchignoni et al. [52], which reported that the 14 items of the Mini-BESTest showed unidimensionality in a heterogeneous neurological sample. The different findings regarding unidimensionality might be the result of a difference in study samples (e.g. heterogeneous clinical samples versus only PwMS), a difference in item characteristics (e.g. focus on ability testing versus focus on exercise performance), and different methodological criteria to confirm unidimensionality.

Analysis showed that targeting of the balance exercises was adequate; i.e. the range of balance exercise difficulty covered the ability estimates of most participants. However, there was a lack of very difficult exercises for dimension 2 “sway” and of easy balance exercises for dimension 3 “step and walk”.

To our knowledge, this is first study to evaluate the targeting of balance exercises in PwMS. These data might help to improve the effectiveness of balance exercises in PwMS by enabling better targeting of exercises to participants’ abilities. Several RCTs have analysed the effectiveness of balance exercises in PwMS [15]. Some studies aimed to tailor the difficulty of balance exercises to the participants’ ability, with the selection of exercises based on clinical reasoning. For example, Cattaneo et al. [20] chose motor and sensory training modalities based on the individual’s abilities. To support health professionals in the selection of exercises the difficulty of balance exercises should be presented on a clear progression line. In addition to an unambiguous progression in exercise difficulty, the distance in difficulty between the exercises should be stated clearly. This could facilitate a more objective allocation of exercises.

The physiotherapists of the expert round ranked the exercises regarding the difficulty similar to the ranking based on the Rasch measures (logits), however there were some discrepancies. For example, in dimension 2 there was a larger discrepancy in the exercise e11 “rolling ball forwards”. This may be because the difficulty of the exercise varies greatly depending on how it is performed, i.e. whether the ball is used as a base of support or rolled forward without pressure. Therefore, the standardisation and instructions for the exercises need to be improved.

### Limitations

This study has several limitations. First, during the process of establishing the content validity of the balance programme quantitative methods were used, such as surveys. No qualitative research methods were used to establish content validity. In addition, the sample size of the expert round (13 physiotherapists) was relatively small, and did not include other professions, such as sports scientists. In contrast, 65 PwMS provided data for the content validity of the balance exercises.

A further limitation was the relatively low number of balance exercises integrated into the Rasch analysis. This was based on pragmatic reasoning; the PwMS should be able to complete the set of balance exercises within a single session. Adequate structural validity was reported for these 19 exercises, which can be used as “key exercises” in the balance programme, while clinicians can also integrate modifications to increase the difficulty of each exercise. For example, by incorporating additional head, eye, arm, leg or trunk movements, a change in surface conditions, dual tasks, or reduced visual information.

A further potential limitation is the focus of the balance exercises. Cattaneo et al. [20] reported on two different balance exercises programmes (training of motor or sensory strategies). Within the current study, the exercises can be classified into the category “training of motor strategies”. Therefore, this study could not report on the difficulty of exercises that alter the sensory environment. We propose to integrate such modifications into the balance exercises with the aim of increasing their difficulty. For example, exercise e6 “tandem stance” has a difficulty of 1.99 logits. If PwMS are asked to perform this exercise with eyes closed or on an unstable surface, the difficulty will increase and the exercise will be more challenging. However, further research is needed to determine by precisely how much the difficulty will increase.

An additional limitation was identified in analysing the threshold values of the balance exercises. The difference in logits between thresholds 1, 2 and 3 were very small. This was also observed during the measurement sessions. For some participants it was challenging to score the difficulty of the exercises. In particular, the categories “very easy” and “easy” were difficult to separate. The scoring system of the subjective difficulty should be investigated further and adapted in future studies. A possible solution would be to combine these options, although this method is controversial. This was not done in the current study because some authors suggest that data should be re-measured after changes to the scoring system [53].

Furthermore, the local dependency (i.e. high residual correlations) of some items (especially in dimension 2) needs to be addressed in the future development of this balance exercise programme. We were not able to precisely estimate the local dependency within our data set, because less than the required 20 exercises were analysed together within one dimension [40].

Dimension 2 showed a low person separation index and reliability. However, this is probably due to the low number of items (*n* = 4) [45] and in line with the standardised reliability (i.e. reliability standardised on 50 items), which was 0.92. More items are needed to increase the ability to distinguish between low and high performers in dimension 2.

There were disordered categories in two dimensions. Possible reasons for disordering are: a) the low counts in the categories could lead to random errors or idiosyncratic findings, b) there could be a problem with the rating scale, e.g. difficulty to understand the meaning of the response options. Since the response options were the same for all items, but the disorder was not consistent, we believe that the low number of observations, which increase the standard error of the estimates, was responsible for the problem [54]. Furthermore, because only three categories showed disordered categories and because we would like to keep the response options the same over all exercises, we did not perform a recoding of these categories.

A limitation in the current study was that differential item functioning was not analysed. This analysis was not done because the sample size was not large enough to conduct an adequate analysis. Further research is needed to explore whether exercise progression might be different in specific subgroups. For example, Sosnoff et al. [55] reported that fall risk differed between groups of PwMS. Factors such as cerebellar or brain stem lesions increased the fall risk. Similarly, PwMS with impaired visual function showed greater balance impairments. Therefore, a larger well-powered study should investigate whether the exercise progression is comparable between these subgroups, or if the balance programme should be modified for each subgroup.

### Implications for practice

The proposed balance programme is one aspect of a multicomponent rehabilitation programme aiming to decrease fall risk in PwMS [15]. However, as balance impairments are reported to precede mobility impairments in PwMS [13], it can be assumed that a targeted balance exercise programme is especially valuable for this population. The 19 key exercises described here can be used as the basis for an extensive home-based exercise programme. The established difficulty estimates for each balance exercise can be used by health professionals to identify the optimal challenge point for training of balance abilities in PwMS. To increase the challenge, each exercise can be modified as follows: arm movements (slow and fast in different planes), trunk and head movements, eyes closed, or addition of secondary motor or cognitive tasks (dual task). Furthermore, different surface conditions can be used (from stable to unstable). The exercises, including adaptations to the difficulty levels, could be implemented via web or tablet applications, including videos and more detailed instructions, and with the option of gathering feedback on difficulty from patients.

### Implications for research

The findings of this study should be investigated further in larger studies. In addition, the response options for the difficulty estimates should be modified, due to the limited information about difficulty on several threshold values (i.e. the difference between thresholds 1–3 was very small). A possible solution would be to combine these response options.

An interesting approach for further research would be to assess the difficulty levels of all exercises (including the adaptations) and to develop recommender systems analogous to computer adaptive testing. After each rating of exercise difficulty, the computer would suggest the most appropriate exercise to the PwMS.

In addition, differential item function should be evaluated in potential subgroups, such as PwMS with or without cerebellar lesions, spasticity and different forms of MS (e.g. primary progressive MS or relapsing-remitting MS).

## Conclusion

This study presents evidence to support the content and structural validity of a balance exercise programme for PwMS. The study initially considered four dimensions of balance exercises in PwMS, but the analysis showed better fit to a 3-dimensional solution, which is in agreement with categories of balance problems in PwMS published recently by others [8, 10]. All proposed balance exercises in the current study demonstrated adequate fit to the dimensions. The identified estimates of difficulty will enable clinicians to target balance exercise difficulty to balance abilities within each dimension.

## Supplementary Information

**Additional file 1.** An example balance exercise (exercise e1). File format: pdf. This webpage was shown to the participants and comprised a photo of the exercise, written instructions and the self-reported scale to rate the difficulty of the exercise.

**Additional file 2.** Pool of 98 balance exercises. File format: xlsx. 98 balance exercises were identified after the literature search. Two authors (KMS, LND) classified involved balance components (such as stability limits, ankle strategy or static balance) indicated with an x.

**Additional file 3.** The 19 key balance exercises and ratings of balance dimensions and mean rank position of exercise difficulty. File format: pdf. The following information is presented: the exercise ID; the exercise name; photo of the exercise; specification in which categories the exercise has been sorted; frequency of ratings within the category; mean rank position; exercise instruction.

**Additional file 4.** Similarity matrix. File format: xlsx. The number of times a pair of exercises were grouped together and the percentage of participants agreeing with an exercise pairing are shown.

**Additional file 5.** Category disordering. File format: pdf. Average (mean) ability estimates are presented for each category. Disordered categories are highlighted with an *.

**Additional file 6.** Correlation expert ratings and Rasch measure. File format: pdf. For each exercise the mean rank position of the expert round (i.e. physiotherapists’ ratings) is correlated with the Rasch measure. The four dimensions (i.e. “Stable BOS”, “Sway”, “Step” and “Walk”) are presented separately. Rank positions are indicated as E (expert round rank) and R (Rasch rank). For example, R1-E2 indicates Rasch 1st rank and expert round 2nd rank.

## Data Availability

The dataset analysed during the current study is available in the Figshare repository, 10.6084/m9.figshare.13525553

## Abbreviations

ADL: Activities of daily living
BOS: Base of support
CI: Confidence interval
CoM: Centre of mass
EDSS: Expanded Disability Status Scale
PCA: Principal component analysis
PwMS: People with Multiple Sclerosis
