# Supplementary material for: Development of an exercise programme for balance abilities in people with multiple sclerosis: a development of concept study using Rasch analysis
Source: Arch Physiother. 2021 Dec 15;11:29. doi: 10.1186/s40945-021-00120-3 (PMC8672542; doi:10.1186/s40945-021-00120-3)
Supplement: Supplementary file 1 — Additional file 1. An example balance exercise (exercise e1). File format: pdf. This webpage was shown to the participants and comprised a photo of the exercise, written instructions and the self-reported scale to rate the difficulty of the exercise. [file 40945_2021_120_MOESM1_ESM.pdf]

Exercise 1:

Assign the probability that you would fall during the exercise shown.

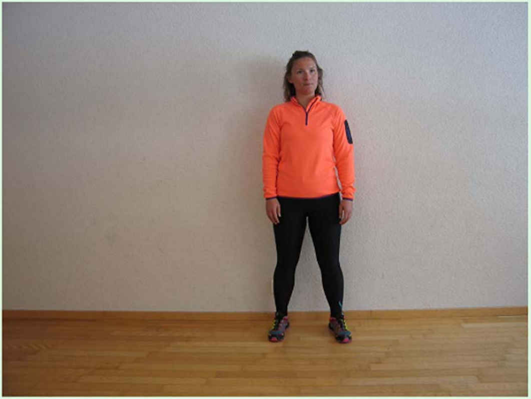

- ○ certainly
- ○ almost certainly
- ○ probably
- ○ 50:50 / about even
- ○ probably not
- ○ almost certainly not
- ● certainly not

reset

Stand for about 15 seconds with your feet wide apart without moving.

Please perform the exercise shown above for 15 seconds and then rate the difficulty of the exercise. If you find the exercise dangerous for you, please do not perform this exercise.

- ○ very easy
- ● easy
- ○ challenging
- ○ very challenging
- ○ too challenging, it is almost dangerous
- ○ dangerous, I would or could fall

reset
