# Supplementary material for: Development of an exercise programme for balance abilities in people with multiple sclerosis: a development of concept study using Rasch analysis
Source: Arch Physiother. 2021 Dec 15;11:29. doi: 10.1186/s40945-021-00120-3 (PMC8672542; doi:10.1186/s40945-021-00120-3)
Supplement: Supplementary file 3 — Additional file 3. The 19 key balance exercises and ratings of balance dimensions and mean rank position of exercise difficulty. File format: pdf. The following information is presented: the exercise ID; the exercise name; photo of the exercise; specification in which categories the exercise has been sorted; frequency of ratings within the category; mean rank position; exercise instruction. [file 40945_2021_120_MOESM3_ESM.pdf]

Exercise ID	Exercise name	Exercise photo	Sorted into categories	Frequency	Mean rank position	Exercise instruction
e1	Standing wide stance	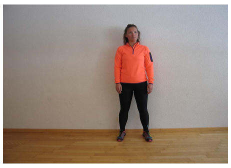	Stable BOS	13	1.1	Stand for about 15 seconds with your feet wide apart without moving.
e2	Standing feet together	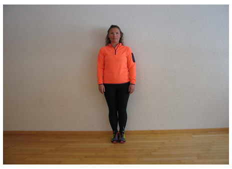	Stable BOS	12	3.2	Stand with your feet together for about 15 seconds without moving.
			Sway	1	3	
e3	Step stance wide feet position	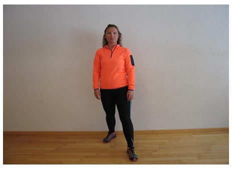	Stable BOS	11	2.2	Stand for 15 seconds with the foot position shown without moving.
			Step	1	1	
			Walk	1	1	
e4	Semi-tandem stance	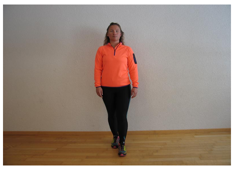	Stable BOS	13	4.4	Stand for 15 seconds with the feet in front of each other and staggered sideways so that the big toe of the back foot touches the inner edge of the heel of the front foot.
e5	Tandem stance feet apart	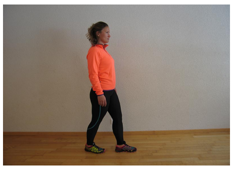	Stable BOS	13	4.3	Stand for 15 seconds with the foot position shown (feet in line, one hand width between the toes of the back foot and the heel of the front foot) without moving.
e6	Tandem stance	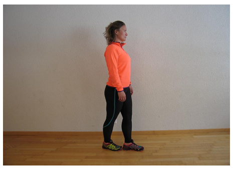	Stable BOS	12	5.6	Stand for 15 seconds with the foot position shown (feet in line, toes and heel touching) without moving
			Sway	1	2	
e7	One-leg stance	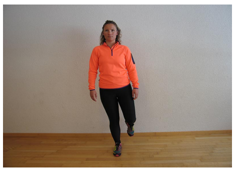	Stable BOS	11	6.9	Stand for 15 seconds with the foot position shown without moving.
			Sway	1	3	
			Step	1	3	
e8	Wall-leaning forwards	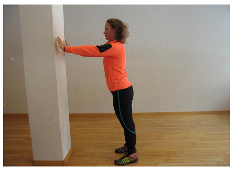	Sway	10	1.7	Stand in front of a wall so that with your arms outstretched there is about 20 cm of space between your fingertips and the wall. Now move your hands as far forward as possible without touching the wall and without losing your balance. Just before the hands touch the wall return to the original position.
			Stable BOS	1	3	
			Step	1	1	
e9	Wall-leaning backwards	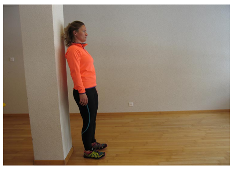	Sway	11	3.5	Stand with your back to a wall so that there is about 10 cm of space between you and the wall. Now move your back towards the wall without touching the wall and without losing your balance. Just before the back touches the wall return to the original position.
			Stable BOS	1	6	
			Walk	1	2	

e10	Standing moving body sideways	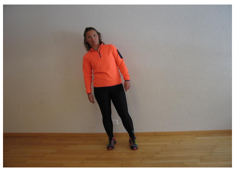	Sway<br>Walk<br>Step	11<br>1<br>1	1.6<br>2<br>1	Stand with the foot position shown and slowly move the whole body to one side as far as you safely can without losing balance. Then return to the original position.
e11	Rolling ball forwards	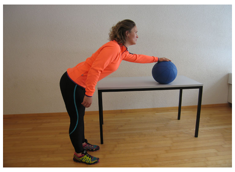	Sway<br>Step	11<br>2	2.7<br>2.5	Stand at the side of a table. Place one hand on the ball on the table without supporting yourself on the ball. Now move the ball forward as far as possible without losing your balance.
e12	Stepping forwards	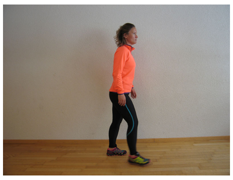	Step<br>not standardized<br>Walk	11<br>1<br>1	1.4<br>1<br>1	First stand on both feet. Then take a single step forward and remain standing on both feet in step position.
e13	Stepping sideways	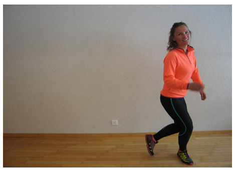	Step<br>Walk	9<br>4	2.9<br>4	First stand on both feet. Now take a sideways step by crossing the right leg in front over the left leg. Continue with the the back leg until you are standing on both feet again.
e14	Stepping backwards	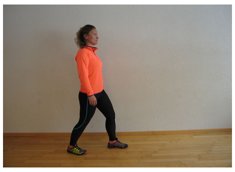	Step<br>Walk	12<br>1	2.8<br>5	First stand on both feet. Then take a single step backwards and stand still on both feet.
e15	Leaning forwards reactive step	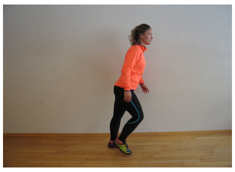	Step	13	3.3	First stand on both feet. Move the whole body forward until you have to take a step in order not to lose your balance. Stand still again on both feet.
e16	Line walking	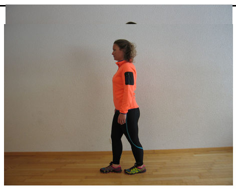	Walk<br>Step	12<br>1	3.1<br>4	First stand on both feet. Then walk forward as if you were walking on an imaginary string. Place your heel just in front of your toes each time.
e17	Walk backwards	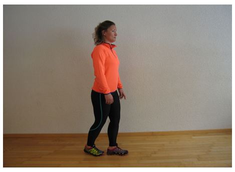	Walk<br>Step<br>Sway	11<br>1<br>1	3.2<br>6<br>1	First stand with both feet side by side. Then walk backwards on an imaginary line. Place your toes just behind your heels.
e18	Heel walking	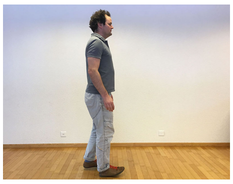	Walk<br>Sway	12<br>1	3<br>3	Walk forward on your heels.
e19	Forefoot walking	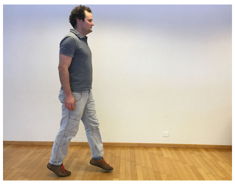	Walk<br>Sway	12<br>1	1.9<br>2	Walk forward on your toes.
