# Supplementary material for: Development of an exercise programme for balance abilities in people with multiple sclerosis: a development of concept study using Rasch analysis
Source: Arch Physiother. 2021 Dec 15;11:29. doi: 10.1186/s40945-021-00120-3 (PMC8672542; doi:10.1186/s40945-021-00120-3)
Supplement: Supplementary file 5 — Additional file 5. Category disordering. File format: pdf. Average (mean) ability estimates are presented for each category. Disordered categories are highlighted with an *. [file 40945_2021_120_MOESM5_ESM.pdf]

Additional information 4: Category disordering

Dimension 1

ENTRY NUMBER	DATA CODE	SCORE VALUE	DATA COUNT	%	ABILITY MEAN	P.SD	S.E. MEAN	INFT MNSQ	OUTF MNSQ	PTMA CORR.	ITEM
7	0	0	27	42	-.46	1.61	.32	1.5	2.6	-.63	e7
	1	1	2	3	.60	.14	.14	.1	.0	-.04	
	2	2	10	15	1.47	.82	.27	.8	.8	.11	
	3	3	15	23	1.48	1.07	.29	1.2	1.1	.14	
	4	4	10	15	3.34	1.04	.35	.7	.6	.52	
	5	5	1	2	4.98	.00		.5	.5	.26	
6	0	0	23	35	-.70	1.51	.32	.8	.7	-.65	e6
	1	1	3	5	.41	.56	.40	1.2	.3	-.07	
	2	2	7	11	.70	.35	.14	.7	.3	-.05	
	3	3	20	31	1.42	.61	.14	.6	.5	.15	
	4	4	10	15	3.77	.84	.28	.2	.3	.62	
	5	5	2	3	4.07	.91	.91	1.2	1.2	.29	
5	0	0	23	35	-.62	1.55	.33	1.0	1.0	-.62	e5
	1	1	4	6	.13	.82	.47	1.1	.8	-.12	
	2	2	1	2	-.08*	.00		4.1	1.8	-.07	
	3	3	22	34	1.13	.46	.10	.7	.5	.05	
	4	4	11	17	3.37	1.01	.32	.4	.5	.56	
	5	5	4	6	4.03	.67	.39	1.0	1.1	.41	
4		***	1	2#	.29	.00				-.05	e4
	0	0	8	13	-2.20	1.35	.51	.4	.3	-.63	
	1	1	2	3	-1.75	.65	.65	.3	.7	-.26	
	2	2	2	3	.02	.10	.10	.3	.2	-.09	
	3	3	25	39	.74	.54	.11	.4	.5	-.11	
	4	4	20	31	1.85	1.10	.25	.7	.8	.30	
	5	5	7	11	4.24	.60	.25	.4	.4	.59	
3	0	0	4	6	-2.64	2.15	1.24	8.5	10.0	-.49	e3
	1	1	1	2	-2.39	.00		.2	.1	-.22	
	3	3	10	15	.45	.68	.23	1.6	1.3	-.12	
	4	4	25	38	.59	.95	.19	1.0	.6	-.16	
	5	5	25	38	2.31	1.77	.36	1.4	1.2	.55	
2	0	0	4	6	-3.36	.94	.55	3.2	3.7	-.58	e2
	2	2	1	2	-2.39	.00		.0	.0	-.22	
	3	3	7	11	-.41	.72	.30	.7	.6	-.25	
	4	4	26	40	.90	.85	.17	1.0	.7	-.04	
	5	5	27	42	2.20	1.64	.32	1.2	1.2	.53	
1	3	3	2	3	-1.76	2.65	2.65	2.9	6.3	-.26	e1
	4	4	13	20	-.70	1.68	.49	.9	.9	-.44	
	5	5	50	77	1.54	1.55	.22	1.0	1.0	.52	

* Average ability does not ascend with category score
# Missing % includes all categories. Scored % only of scored categories

Dimension 2

ENTRY NUMBER	DATA CODE	SCORE VALUE	DATA COUNT	%	ABILITY MEAN	P.SD	S.E. MEAN	INFT MNSQ	OUTF MNSQ	PTMA CORR.	ITEM
2	0	0	4	6	-.92	.32	.18	.4	.3	-.40	e9
	1	1	1	2	-.55	.00		.0	.0	-.18	
	2	2	4	6	.15	.30	.17	.2	.1	-.29	
	3	3	14	22	1.27	1.33	.37	1.7	2.5	-.34	
	4	4	37	57	3.58	1.55	.26	.7	.9	.36	
	5	5	5	8	7.50	.63	.32	.5	.4	.56	
1	2	2	2	3	-.37	.48	.48	1.2	.8	-.24	e8
	3	3	16	25	.97	1.15	.30	1.2	1.1	-.44	
	4	4	33	51	2.45	1.60	.28	2.6	1.1	-.16	
	5	5	14	22	6.32	1.17	.33	.5	.5	.76	
3	0	0	2	3	-1.56	.13	.13	.9	.8	-.32	e10
	3	3	5	8	.40	1.10	.55	1.5	1.1	-.29	
	4	4	36	55	1.98	1.52	.26	.9	1.0	-.39	
	5	5	22	34	5.17	1.82	.40	1.0	1.1	.70	
4		***	1	2#	1.40	.00				-.07	e11
	0	0	1	2	-1.69	.00		.8	.8	-.24	
	3	3	4	6	-.47	.25	.15	.8	.5	-.35	
	4	4	34	53	1.87	1.40	.24	.8	.6	-.43	
	5	5	25	39	4.91	2.04	.42	1.2	1.1	.68	

Missing % includes all categories. Scored % only of scored categories

Dimension 3

ITEM CATEGORY/OPTION/DISTRACTOR FREQUENCIES: MEASURE ORDER

ENTRY NUMBER	DATA CODE	SCORE VALUE	DATA COUNT	%	ABILITY MEAN	P.SD	S.E. MEAN	INFT MNSQ	OUTF MNSQ	PTMA CORR.	ITEM
6		***	1	2#	1.71	.00				.12	e17
	0	0	34	53	-1.16	1.14	.20	.6	.5	-.75	
	1	1	1	2	-.71	.00		2.5	.7	-.06	
	2	2	9	14	.64	.47	.17	.6	.2	.14	
	3	3	13	20	1.45	.87	.25	.9	.6	.42	
	4	4	6	9	2.57	1.06	.47	.6	.6	.48	
	5	5	1	2	2.62	.00		1.1	1.3	.19	
5	0	0	26	40	-1.30	1.18	.24	1.0	.9	-.65	e16
	1	1	3	5	-1.43*	.71	.51	1.4	3.4	-.19	
	2	2	7	11	-.24	.48	.20	.9	.6	-.06	
	3	3	17	26	1.06	.91	.23	.8	.9	.35	
	4	4	9	14	1.74	.57	.20	.4	.5	.39	
	5	5	3	5	3.42	.61	.43	.6	.6	.43	
7	0	0	26	40	-1.30	1.35	.27	1.7	3.5	-.65	e18
	1	1	4	6	-.62	.65	.38	.6	.6	-.10	
	2	2	2	3	.97	.74	.74	1.6	1.5	.09	
	3	3	15	23	.44*	.68	.18	1.1	.9	.12	
	4	4	14	22	1.61	1.23	.34	1.5	1.4	.47	
	5	5	4	6	2.36	.72	.41	1.1	1.2	.34	
8	0	0	18	28	-1.85	1.11	.27	1.1	1.4	-.69	e19
	1	1	1	2	-.71	.00		.6	.3	-.06	
	2	2	6	9	-.46	.34	.15	.4	.2	-.10	
	3	3	13	20	.10	.78	.23	.9	.7	.01	
	4	4	16	25	.91	.84	.22	.8	.7	.28	
	5	5	11	17	2.29	1.09	.34	1.0	1.1	.59	
4	0	0	14	22	-1.91	1.25	.35	1.5	2.2	-.61	e15
	1	1	1	2	-1.63	.00		.1	.1	-.12	
	2	2	5	8	-.44	1.04	.52	1.7	1.6	-.09	
	3	3	19	29	-.11	.89	.21	1.3	1.3	-.07	
	4	4	18	28	1.08	1.04	.25	.8	.8	.37	
	5	5	8	12	2.18	1.28	.48	1.1	1.1	.46	
2	0	0	12	18	-2.16	1.10	.33	1.4	1.4	-.62	e13
	1	1	2	3	-2.12	.48	.48	.2	.4	-.23	
	2	2	1	2	-.95	.00		.2	.1	-.07	
	3	3	16	25	-.32	.77	.20	.6	.7	-.13	
	4	4	21	32	.52	.68	.15	.6	.5	.19	
	5	5	13	20	2.27	1.08	.31	.8	.9	.65	
3	0	0	3	5	-3.43	1.01	.72	2.0	1.7	-.45	e14
	1	1	1	2	-2.60	.00		.1	.1	-.20	
	2	2	4	6	-2.28	.52	.30	.5	.3	-.35	
	3	3	7	11	-1.01	.67	.27	.7	.7	-.22	
	4	4	34	52	.15	1.06	.18	.7	1.1	.05	
	5	5	16	25	1.76	1.22	.31	.9	.8	.57	
1	0	0	3	5	-3.79	.50	.35	.3	.2	-.50	e12
	2	2	2	3	-1.54	1.06	1.06	1.1	1.7	-.17	
	3	3	9	14	-1.17	.79	.28	.7	.7	-.29	
	4	4	32	49	.09	1.08	.19	1.0	.9	.02	
	5	5	19	29	1.39	1.56	.37	1.4	1.2	.50	

* Average ability does not ascend with category score
# Missing % includes all categories. Scored % only of scored categories
