# Supplementary figures and images for: Development of an exercise programme for balance abilities in people with multiple sclerosis: a development of concept study using Rasch analysis
Source: Arch Physiother. 2021 Dec 15;11:29. doi: 10.1186/s40945-021-00120-3 (PMC8672542; doi:10.1186/s40945-021-00120-3)

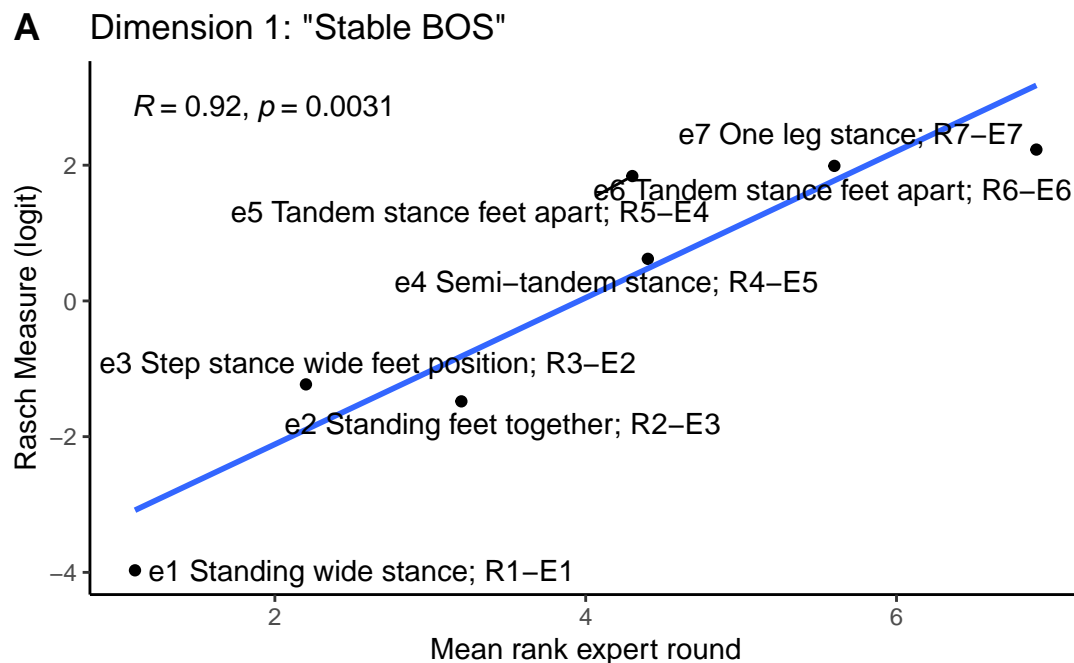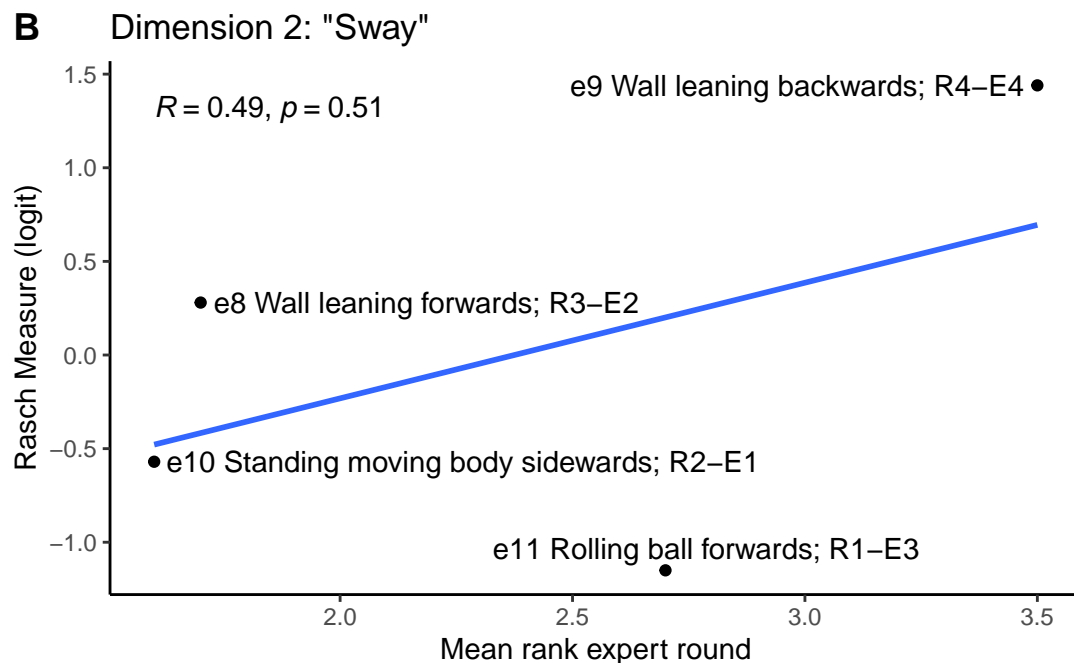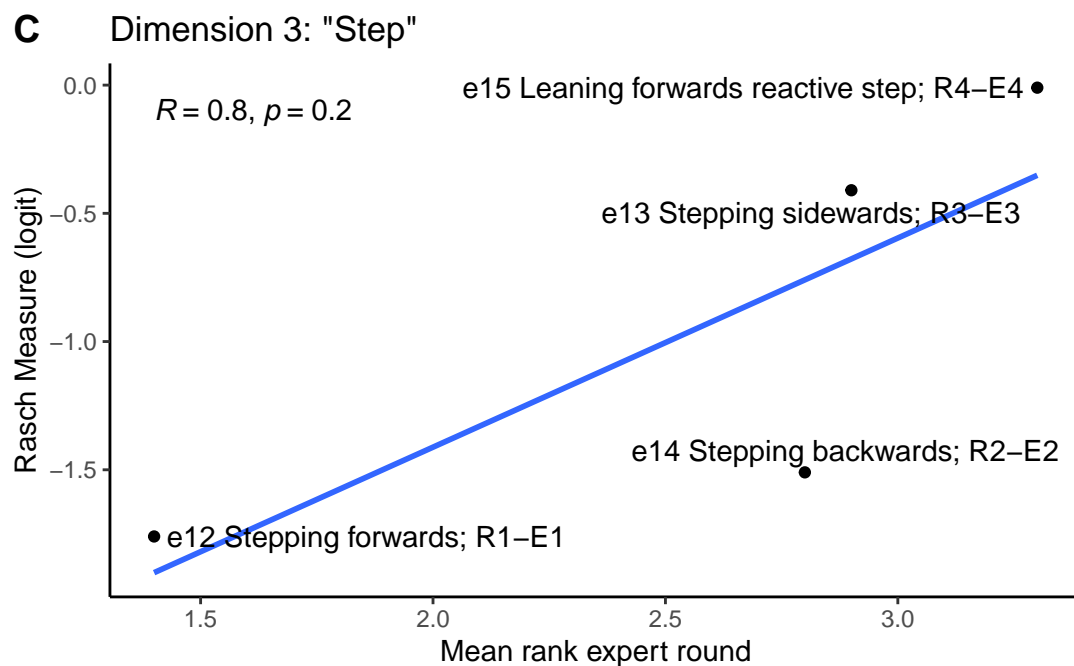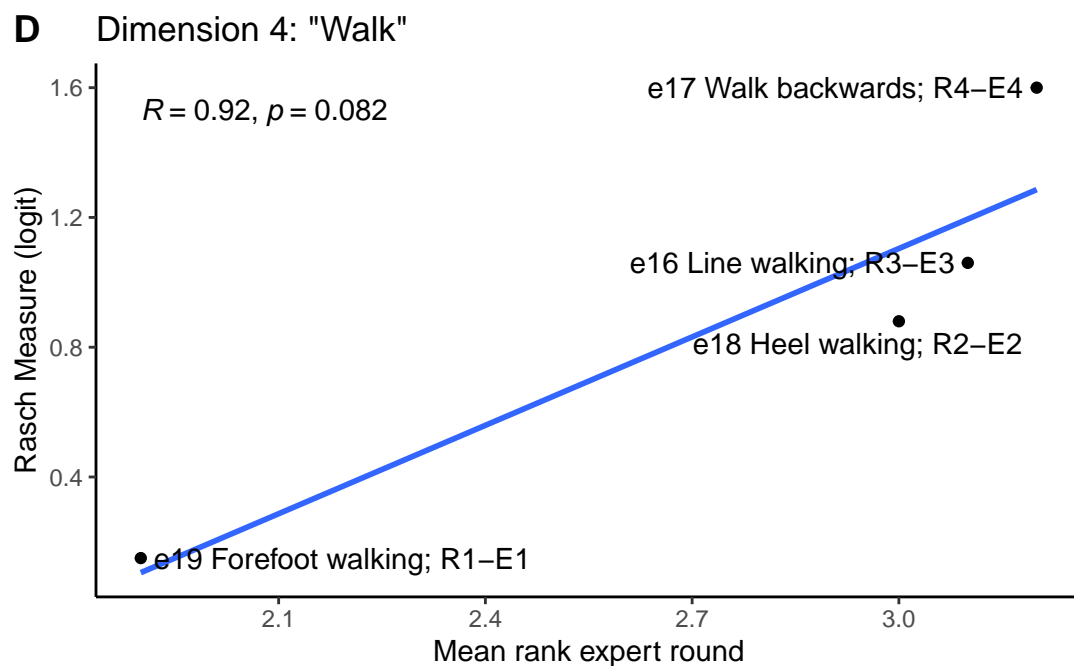

Supplement: Supplementary file 6 — Additional file 6. Correlation expert ratings and Rasch measure. File format: pdf. For each exercise the mean rank position of the expert round (i.e. physiotherapists’ ratings) is correlated with the Rasch measure. The four dimensions (i.e. “Stable BOS”, “Sway”, “Step” and “Walk”) are presented separately. Rank positions are indicated as E (expert round rank) and R (Rasch rank). For example, R1-E2 indicates Rasch 1st rank and expert round 2nd rank. [file 40945_2021_120_MOESM6_ESM.pdf]
